# The Effectiveness of a Question-Embedded Movie Clips Learning Program in Nursing Students: A Quasi-Experimental Pretest-Posttest Study

**DOI:** 10.2196/71111

**Published:** 2025-07-08

**Authors:** Chanokruthai Choenarom, Juraipon Samputtanon

**Affiliations:** 1 Faculty of Nursing, Khon Kaen University, 123 Mittraphap Road, Khon Kaen, 40002, Thailand, 66 93-080-3031

**Keywords:** web-based, embedded questions, video clips, therapeutic communication, nurse-patient relationship, nursing students

## Abstract

**Background:**

Technological innovations make significant impacts on nursing education. New teaching strategies are constantly emerging, offering students a dynamic and interactive educational experience. The Question-Embedded Movie Clips Learning program used in this study was developed based on the drill and practice learning principle, aiming to facilitate nursing students’ skills and build their confidence before entering real clinical settings.

**Objective:**

This study aims to investigate the comparative effect between the newly developed Question-Embedded Movie Clips Learning program and the current practice of mind mapping exercises on students’ learning outcomes and study satisfaction.

**Methods:**

This study adopted a quasi-experimental design using a pretest-posttest approach with nonequivalent groups. The study sample consisted of 132 third-year nursing students who enrolled in one of two class sections of the psychiatric nursing course at a university in Thailand. By flipping a coin, the first study section (n=62) was assigned to the intervention, and the second section (n=70) was assigned to the control group. During the 2-hour class sessions, students received identical learning structure and sequence, except for group exercises of either the Question-Embedded Movie Clips Learning program or the mind mapping. The data were collected through the pretest-posttest questionnaire, the perceived satisfaction with the learning experience scale, and the open-ended reflective questions.

**Results:**

A statistically significant increase was observed in the learning outcome scores of both the intervention group (*t*_61_=–30.48, 95% CI –10.59 to –9.28; *P*<.001) and the control group (*t*_69_=–27.04, 95% CI –8.19 to –7.07; *P*<.001); all *t* statistics reported are based on 2-tailed tests. There was, however, a statistically significant difference in the outcome scores between the 2 groups. Even after controlling for pretest scores, students in the experimental group had a significantly higher adjusted mean score than those in the control group (*F*_1,129_=67.67, *P*<.001).

**Conclusions:**

The study has provided empirical evidence that using the Question-Embedded Movie Clips Learning program along with traditional instruction in teaching therapeutic relationships and communication significantly improves learning outcomes.

## Introduction

### Background

An important skill set for nurses is interpersonal skills and communication. Mental health, in particular, is a branch of nursing that applies the therapeutic use of self and relationships as a means of bringing about positive health changes in patients. Despite being crucial for nursing care, many nursing students find learning interpersonal skills to be a challenge and struggle to establish therapeutic relationships with patients. As not all attributes of these complicated skills can be taught in lecture classrooms, nursing students express having difficulty learning them through conventional instruction and show preferences for more innovative, interactive, and practice-based strategies [1,2]. Nursing educators, therefore, need to shift from familiar teacher-centered teaching toward student-centered learning to provide an impactful educational experience.

Among studies aiming to identify the best teaching methods for clinical skill acquisition, there seems to be a consensus emerging about the importance of active learning and student engagement. Cumulative evidence strongly supports the benefit of incorporating instruction that is self-directed, reflective, immediate, stimulating, and experientially immersive to produce a constructive impact on students’ learning outcomes [3]. Pedagogical strategies that carry labels such as student-directed education, process-oriented instruction, or experiential and authentic learning are increasingly being explored by nurse educators. As a result, innovative teaching, including simulation-based learning, flipped classrooms, artificial intelligence–based training, and web-based learning platforms with augmented or virtual reality are gradually being embraced as methods to prepare nursing students to meet professional demands [4,5].

Learning therapeutic communication effectively involves tailoring instruction to different learning styles and using a variety of teaching methods. By acknowledging and supporting learning preferences, student engagement and desirable learning outcomes can be ensured. Recent review studies highlighted the preference of kinesthetic and multimodal to be the most dominant learning style among undergraduate nursing students [6-8]. Several studies have also explored the issue of learning styles among Thai nursing students. A descriptive study examined the learning styles of 177 Thai nursing students using the Felder and Solomon index and found that most students (89.8%) were sensing learners who prefer to learn from observation, action, and problem-solving in gradual stages [9]. The result corresponded with studies reporting the majority of Thai nursing students’ preference for multimodal learning of visual, auditory, read or write, and kinesthetic learning [10-12] with participative learning style being most prevalent [13]. Accordingly, this study incorporates movie-based case studies, embedded practice questions, and group work for learning strategies. Movie clips were used as real-world examples to promote critical thinking and reinforce practical skills through problem-solving. Additionally, group work and in-class presentations can promote active involvement, peer discussions, and knowledge sharing.

In Thailand, 7 studies on the use of innovative teaching strategies were found particularly relevant to teaching nursing students therapeutic communication. Two studies using standardized patient simulation reported positive effects on students’ learning outcomes and satisfaction with mental health and psychiatric nursing courses [3,14]. Another 3 studies used computer-assisted instruction to create online lessons and found that students had higher scores on learning achievement, perceived efficacy, and communication skills compared to students in the control group, who received conventional lectures [15-17]. Two of the studies explored using integrated digital technologies to provide an immersive learning experience for teaching therapeutic relationships and management of psychiatric patients in specific situations. The first study used augmented reality applications on smartphones and reported a high level of student satisfaction after the study [18]. The other study that used a virtual reality learning program found that students had higher scores on attitudes and confidence in managing patient aggression [19].

The current format for teaching interpersonal skills and therapeutic communication is text-based lectures combined with mind mapping group exercises. From the 2020-2022 academic year, mind mapping has been used as the standard teaching method for this topic. While exam results are satisfying for all learning requirements, the majority of nursing students who have completed the course feel that they have insufficient knowledge to effectively engage in therapeutic relationships with the patients. These difficulties in transferring theoretical knowledge into clinical practice are viewed as major obstacles in nursing education [20]. As lectures can make learning relatively superficial and transient, active learning strategies are needed. Though innovative strategies are being adopted by Thai nurse educators, the methods are restricted to tutorial and simulation use; other training strategies such as drill and practice and problem-solving instructions have not yet been studied for their effectiveness. To overcome the limitation of traditional text-based teaching, the Question-Embedded Movie Clips Learning (QEMCL) program used in this study was designed based on drill and practice, as well as instructional game principles. The aim of the study is to examine the effectiveness of the QEMCL program on nursing students’ satisfaction and learning outcomes on the therapeutic use of self and communication skills.

### Theoretical Framework

This study used the Classroom Action Research framework based on the Plan-Do-Check-Act cycle to make continuous teaching improvements. The cycle begins with the “PLAN” stage, which deals with preparing class lessons. The “DO” stage involves teaching the lesson, engaging in learning activities, and assigning work. The “CHECK” is to assess learning outcomes and make recommendations for improvements, and the “ACT” stage is the use of the improved teaching strategies in the classroom. Presenting the findings, or outcomes, of this learning project correlates with the “CHECK” stage, which aims to check whether what had been planned in the first stage has been achieved. The recommendations of this study are related to the “ACT” stage, which aims to improve the learning outcome of the lesson [21].

Literature reviews on studies examining the effectiveness of educational interventions for medical and nursing students’ therapeutic communication skills have been widely conducted [22-25]. These reviews, however, primarily focused on summarizing educational programs, while only a few studies included a description of a theoretical framework. A more recent systematic review found that employing Jonassen’s constructivist learning environments (CLEs) [26] can effectively promote the therapeutic communication education of nursing students and, therefore, was recommended for nurse educators [27]. The CLE instructional design postulates that students learn best by solving practical problems to integrate new knowledge into what they already know. Adopting this approach, this study was planned in accordance with CLE elements by providing problem-based case scenarios with the use of videoclips as cognitive tools and lecture clips as related information resources. A modeling instructional method was used, with the movie character acting as a role model illustrating the use of therapeutic communication techniques. With the constructivist approach, the teaching method of this study was designed to encourage critical thinking, problem-solving, and collaborative learning among nursing students.

## Methods

### Ethical Considerations

This study was approved by the ethics committee of Khon Kaen University (no. HE 662147). The participants consisted of nursing students from the Faculty of Nursing at Khon Kaen University. Students were offered the opportunity to join the study voluntarily, with the assurance that their participation would not affect their academic standing or grades. Informed consent was obtained from all study participants. Participants were assigned code numbers for all data entry purposes. The participant-to-code number list was then destroyed after all study data had been collected. No compensation was provided to participants for participating in this study.

### Sample and Setting

Using the effect size of computer-based education on nursing knowledge from an earlier study [28], the power analysis yielded at least 59 participants, with an effect size of 0.43, a significance level of 0.05, and a power of 0.9. The inclusion criteria were (1) being enrolled in the psychiatric nursing course, (2) having access to a device (smartphone, tablet, or computer) that can perform online tasks, and (3) agreeing to participate in the study. The exclusion criteria were retaking the psychiatric nursing course. There were 132 third-year nursing students enrolled in one of two sections of the psychiatric nursing course at Khon Kaen University for the 2023 academic year who met the inclusion and exclusion criteria. For practical reasons and to ensure a comprehensive analysis, all 132 students were recruited to participate in the study on a voluntary basis.

### Recruitment and Data Collection

**Figure 1.**
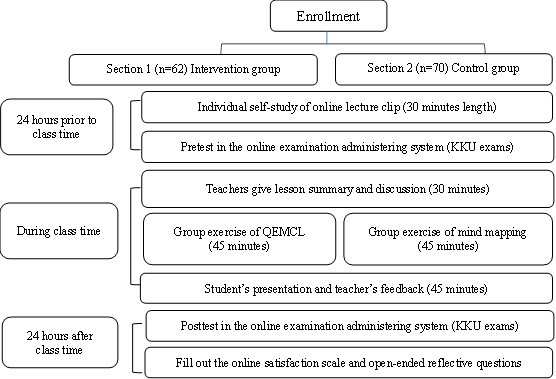
The flowchart of the research procedure. KKU: Khon Kaen University; QEMCL: Question-Embedded Movie Clips Learning program.

The recruitment process started 2 weeks prior to the class date. By flipping a coin, students in the first study section (n=62) were assigned to the intervention, and those in the second section (n=70) were assigned to the control group. In the process of informed consent, students were informed about their potential placement in either an intervention or an active comparison group. Adobe Acrobat Sign was used to obtain online digital signatures of students’ informed consent. By using codes, students’ identities were not directly disclosed to ensure privacy and confidentiality. All students gave their permission to be part of the study and signed their consent to participate. To ensure structural consistency, both groups received a 30-minute self-study online lecture, took the pretest within 24 hours prior to the class session, and participated in a 2-hour class session. The class time for both groups was on the regular learning schedule that runs along the academic semester, so there was no interference with students’ other learning that potentially affected their focus and willingness to engage. During the class sessions, students received identical learning materials and sequences, except for group exercises of either the QEMCL program or mind mapping. Class sessions for both sections were held on the same day, scheduled during consecutive time periods (8‐10 AM for the first study section and 10‐12 PM for the second study section), and led by the same instructor. Students in both groups took the posttest and filled out the satisfaction scale and open-ended reflective questions within 24 hours after class (see Figure 1). All tests were scheduled at the same time using the university’s online examination administering system.

### Instruments

There were 3 instruments used for data collection. The first was the QEMCL program, which is an interactive web-based learning program using question-embedded short movie clips that illustrate therapeutic relationships and communication techniques. This program is composed of short clips from 3 movies, each highly acclaimed for educational use: *Good Will Hunting* [29], *Ordinary People* [30], and *The Prince of Tides* [31]. Researchers selected 7‐8 sequential clips from each movie, corresponding to the typical number of psychotherapy sessions. Each clip had 7‐8 questions embedded in it, making a total of 53‐62 questions per movie. The questions were organized to foster students’ reflective activities, encourage them to identify with characters, and stimulate their clinical judgment. Interactive functions provided in the program to enhance active practice and skill development include immediate feedback with short contextual explanations, real-time scoring, and summative tests with certificates of accomplishment. The content validity of study questions was evaluated by 3 experts in the field, yielding an index of item-objective congruence (IOC) of 0.85. The program had an index of process and output efficiency (E1/E2) of 85/82.6. Details of the QEMCL program development and the evaluation of its efficiency have been reported elsewhere [32].

Only *Good Will Hunting* was selected for this study as the intervention group class exercise due to its coherent run-time and the 2-hour class period limitation. There were 7 selected scenes—Scene 1: First meeting; Scene 2: It’s your turn to move; Scene 3: Super philosophy; Scene 4: Regrets; Scene 5: What do you want to do?; Scene 6: It’s not your fault; and Scene 7: Do what your heart tells you. These scenes were chosen as they capture the essence of how phases of the therapeutic relationship can be constructed through therapy sessions [33]. Scene 1 portrayed the initial phase of the relationship with the important concept of mistrust along with the power of therapeutic authenticity and genuine connection. Scene 2 represented the identification phase where the therapist shows unconditional positive regard, respect, and empathy that creates a safe environment for therapeutic engagement. Scenes 3 through 5 depicted the working phase in which the therapist employs a delicate balance of self-disclosure, compassion, and directness to challenge the client’s defense mechanisms. Scenes 6 and 7 portrayed how the therapist was working toward the termination phase, where masterful uses of active listening, nonjudgmental acceptance, and inducing emotional catharsis are illustrated to help the client explore his experiences, gain insights, overcome his emotional wounds, and find his purpose in life.

As *Good Will Hunting* is an American film, language barriers can be a concern. Additionally, the portrayal of American culture, including its values and beliefs, may be unfamiliar to students from different backgrounds. To overcome these challenges, the Thai subtitles were provided so that students could follow the story efficiently and be able to focus on the characters’ behaviors and intentions. Although cultural differences undoubtedly shape language styles, the interpretation of meaning should not be reduced to stereotypical thinking, but instead to develop awareness, respect, sensitivity, and curiosity to understand value orientations. To facilitate culturally responsive learning, students were asked, as part of group exercises, to identify the scenes they thought showed some aspect of American culture, analyze the different styles of communication, and propose alternative approaches from another cultural perspective. Using these teaching activities, the film can be a valuable resource in helping students recognize culturally influenced behavior and apply the communication strategies depicted in the movie to their own nursing practice.

The second instrument was a 30-item multiple-choice structured pretest-posttest questionnaire developed by the researchers. This questionnaire consists of 3 subscales measuring students’ learning outcomes of memorization, understanding, and applied knowledge. The possible score range was 0 to 30 points. The reliability of the questionnaire was confirmed using the test-retest method with an intraclass correlation coefficient of 0.84. The third instrument was a 10-item scale designed by the researchers to assess students’ perceived satisfaction with the learning experience. Each item featured a 5-point Likert scale ranging from strongly satisfied to strongly dissatisfied with a possible score from 0 to 50. This measure consists of 3 subscales of the learner’s usability, learning content, and the system and function. The pilot test of this scale yielded a Cronbach α coefficient for internal consistency of 0.83. Finally, 2 open-ended reflective questions were used to evaluate students’ intention to recommend learning exercises to others as well as their perceived benefits and limitations of the exercises.

### Statistical Analysis

Data were analyzed with descriptive statistics, chi-square test, *t* test, and analysis of covariance (ANCOVA) using Stata 16.1. To check for ANCOVA assumptions, the homogeneity of regression slopes was examined. A nonsignificant interaction between pretest scores and groups (*P*=.21, 95% CI –0.52 to 0.11) was found, suggesting that the slopes of regression lines for the control and intervention groups were the same. For the homogeneity of variances, Levene test results indicated that the variances were equal across the 2 groups (*F*_1,130_=0.7, *P*=.4). Additionally, the normality of residuals was confirmed by the q-q plot, the kernel density histogram, and the Shapiro-Wilk test (*w*_132_=0.98, *P*=.12).

## Results

Before analyzing the outcome differences, descriptive statistics of each variable were performed. Table 1 presents the results of the homogeneity test for students’ general characteristics. The average ages of students in the intervention and control groups were 21.61 (SD 0.75) and 21.53 (SD 0.58), respectively. There were higher percentages of women than men in both the intervention group (60/62, 97%) and the control group (63/70, 90%). No significant difference, however, was observed in either mean age (*t*_130_=–0.72, two-tailed, 95% CI –0.32 to 0.15; *P*=.47) or proportion of gender (*χ*^2^_1_=2.4, *P*=.12).

**Table 1.** Demographics of the participants in the intervention and control groups.

Variable	Control group (n=70)	Intervention group (n=62)	Statistic test
Age (years), n (%)			*t*_130_=–0.72^a^, 95% CI –0.32 to 0.15; *P*=.47
21	36 (51)	31 (50)	
22	31 (44)	27 (44)	
23	3 (4)	1 (2)	
24	0 (0)	3 (5)	
Mean (SD)	21.61	21.53	
Gender, n (%)			*χ*^2^_1_=2.4, *P*=.12
Male	7 (10)	2 (3)	
Female	63 (90)	60 (97)	

a Independent 2-tailed *t* test.

As shown in Table 2, the learning outcome scores and students’ satisfaction of both groups were compared. The average pretest scores of the intervention group (n=62) and the control group (n=70) were not statistically different (*t*_130_=0.3, 95% CI –0.51 to 0.67; *P*=.77). The *t* statistics reported elsewhere are based on 2-tailed tests. Compared to the control group, the intervention group had a larger increase between pretest and posttest, with significantly different posttest scores (*t*_130_=–8.23, 95% CI –2.75 to –1.68; *P*<.001). The overall satisfaction scores of both the intervention and control groups were high, with each item ranging from 4 to 4.8. There was, however, a significant difference in student satisfaction scores between the 2 groups (*t*_130_=–2.93, 95% CI –3.24 to –0.63; *P*<.001). The students in the intervention and control groups were most satisfied with the learner’s usability (mean 4.52, SD 0.5; mean 4.31, SD 0.51), system and function (mean 4.24, SD 0.51; mean 4.05, SD 0.55), and learning content (mean 4.11, SD 0.44; mean 3.92, SD 0.43), respectively.

**Table 2.** Comparison of the learning outcome and student satisfaction.

Variable	Control group (n=62), mean (SD)	Intervention group (n=70), mean (SD)	Independent *t* test^a^
Learning outcome			
Pretest	15.43 (1.83)	15.34 (1.60)	*t*_130_=0.3, 95% CI –0.51 to 0.67; *P*=.77
Posttest	23.06 (1.41)	25.27 (1.68)	*t*_130_=–8.23, 95% CI –2.75 to –1.68; *P*<.001
Paired *t* test^a^	*t*_69_=–27.04, 95% CI –8.19 to –7.07; *P*<.001	*t*_61_=–30.48, 95% CI –10.59 to –9.28; *P*<.001	
Student satisfaction	40.76 (3.67)	42.69 (3.93)	*t*_130_=–2.93, 95% CI –3.24 to –0.63; *P*<.001

a Two-tailed.

To avoid any potential difference in the respondents’ levels of knowledge at the beginning of the study, tests of ANCOVA were conducted to examine the comparative effects of the QEMCL and mind mapping strategies on student posttest scores, with students’ pretest scores as the covariate. The results indicate that there was a significant difference in learning outcomes between the 2 groups, with *F*_1,129_=67.67, *P*<.001. As shown in Table 3, after controlling for pretest scores, the posttest scores were related to the group variable. Students in the QEMCL group had a significantly higher adjusted mean score (mean 25.27, SD 0.19) than those in the mind mapping group (mean 23.06, SD 0.18). Moreover, the partial eta square (*η*^2^) values provided information about the effects of the pretest and the groups on the posttest scores. The effect size of the group on the posttest was 0.3441, meaning the group variable explained 34.2% (160/465) of the posttest learning outcome after removing the variation explained by the pretest. On the other hand, the effect size of the pretest was 0.0165, which means approximately 1.7% (5.11/310.11) of the posttest variance was explained by the pretest.

**Table 3.** Results of ANCOVA^a^ test for mean scores of learning outcomes.

Variable	Partial sums of squares	Degrees of freedom	Mean square	*F* test	Significance	Partial eta-square
Model	166.72	2	83.34	35.26	.000	0.3534
Groups	160.00	1	160.00	67.67	.000	0.3441
Pretest score	5.11	1	5.11	2.16	.144	0.0165
Residual	305.00	129	2.36	—^b^	—	—
Total	471.72	131	3.60	—	—	—

a ANCOVA: analysis of covariance.

b Not applicable.

As for the question about the students’ intention to recommend learning exercises to other students, 62 (100%) students of the intervention group (n=62) indicated that they would recommend the QEMCL program to others, while 59 (84%) of those in the control group (n=70) did so for the mind mapping activity. In the intervention group (n=62), 54 (87%) students stated that the QEMCL program was highly beneficial to their learning, and 8 (13%) stated that it was somewhat beneficial. In the control group (n=70), only 46 (66%) and 23 (33%) students, respectively, gave the mind mapping exercise the same ratings. No students stated that either learning activity was of small benefit or no benefit at all to their learning.

Table 4 presents key quotations excerpted from the open-ended responses regarding the perceived benefits and limitations of the learning exercises. The benefits included (1) improving learning efficiency, (2) thought provoking, (3) enhancing understanding, and (4) inspiring self-directed learning. The limitation was an inadequate application feature. Students’ reflections on learning experiences also revealed significant implications that teachers should design interactive learning programs. Specifically, students defined the benefits of the program associated with its capacity to foster deep learning and enhance memory content, reduce anxiety, and increase motivation, as well as the simplicity of use. Reported program limitations were associated with the small number of movies and with technical problems.

**Table 4.** Students’ reflections on learning experience.

QEMCL^a^	Mind mapping
Perceived benefits	
Improving learning efficiency	
“Repetitive learning through the exercise helps me to see the same thing from different angles and start to recognize a pattern, and that, for me, brings out clarity.” [ID#0128] “The clips provided context of when, why, and how to use particular communication techniques.” [ID#0156]	“It turns the boring and hard to understand textbook lessons into beautiful artworks that is a lot easier to pay attention to.” [ID#0205]
Thought provoking	
“You don’t know what to say to your patients, until you hear it from the experts. It can, at least, get you start before you can find your own (words).” [ID#0154] “The movie gets me prepared of all the possible challenging situations I might face during the encounter with patients. Especially the violent behaviors at the beginning. Yeah, I think that’s what I get the most from the movie, how to get past all the mistrust before being able to work on real problems.” [ID#0134]	“Comparing your work with those of other groups helps to correct the misunderstandings and highlight importance issues.” [ID#0252]
Enhancing understanding or disputing the misunderstanding	
“I don’t think I can ever understand (the concept of) “abreaction” from reading. Even after seeing it from the movie, it’s still hard to describe in words.” [ID#0151] “The movies give me a realistic picture of therapeutic relationship.” [ID#0107] “Depression or aggressiveness, they all sound the same to me from the lecture, until I saw the clips and had to answer the question, then, I could tell them apart.” [ID#0115] “I used to think that I have to always say smart things, give recommendations for patients to solve their problems, you know, being a superior one for them to depend on. But Dr. McGuire did the opposite, he had so many chances to tell Will what is the right thing to do, but he didn’t. Instead, he asked and waited for the answer from Will, even when Professor Lambeau asked him to tell Will what to do. That makes me realized that I was wrong, and I need to figure out what is actually the purpose of therapeutic communication.” [ID#0134]	“By trying to identify key concepts, sorting them into categories, and linking them together to get a comprehensive picture, the class content has been simplified so that it is easy to understand and memorize.” [ID#0221] “Pictorial illustrations make abstract concepts perceptible and manageable.” [ID#0256] “Mind maps help connect concepts and makes the dispersed detailed become one conforming theme.” [ID#0260]
Promoting culturally responsive learning	
“I think grabbing patient’s neck is not an acceptable manner for the therapist to do, but that might happen to anybody when you lost the temper, even for therapist, whether he’s an American or Thai.” [ID#0107] “I feel that the way Will talk to Dr. McGuire is not what I normally see in Thai patients. It’s almost like talking back to someone you should pay more respect to.” [ID#0142]	—^b^
Inspiring self-directed learning	
“Immediate feedback and scoring make me learn faster, it’s like you have a personal tutor.” [ID#0124]	“You can keep the (mind map) work for review before exam.” [ID#0229]
Perceived limitations	
Limited application features	
The complexity of the infrastructure “Some of the provided explanations are too short. It’ll be better to elaborate to some extent. Or even better, add the function that we (students) can ask for more explanation if needed.” [ID#0157]	—

a QEMCL: Question-Embedded Movie Clips Learning.

b Not applicable.

## Discussion

### Principal Findings

The results of this study showed increased posttest scores for both the QEMCL (intervention) and mind mapping exercise (control) groups. The implementation of both learning methods is, therefore, supported for improving students’ learning outcomes on therapeutic use of self and communication. However, there was a significant difference between the posttest scores of the 2 groups after controlling for the effect of pretest scores. The difference was in favor of the experiment group, indicating a higher learning achievement by the QEMCL group (*P*<.001). The effect size of 0.3441, as measured by partial eta square, was quite large, with 34.4% (160/465) of the difference in variance explained by overall learning. This magnitude of effect is consistent with previous meta-analysis studies, which have shown positive computer-based education effects on knowledge ranging from 0.24 to 0.43 [28,34]. The results suggest that QEMCL can be used as a teaching tool to improve upon traditional teaching strategies.

Both groups showed significant differences between pretest and posttest scores, suggesting that the 2 approaches are both powerful instructional tools for nurse educators. This finding is in line with previous studies that support the benefit of using mind mapping and film-based learning strategies. The focus on classification and memorization makes the mind mapping exercise particularly beneficial to students’ ability to process and absorb new knowledge [35]. As students create mind maps, they learn to retrieve keywords, conceptualize learning content, and organize their thinking to generate comprehensive illustrations [36,37]. Moreover, students’ enjoyment of generating artistic drawings facilitates their emotional engagement and memory retention [38,39]. Similarly, the movie clips in the QEMCL program enable students to envision themselves engaging in therapeutic relationships with patients. The exercise provides visual tools that help students learn by watching the clips, and then analyzing and exchanging their thoughts with others. This art-based learning method has been described as a Visual Thinking Strategy (VTS). By stimulating visual areas of the brain, the VTS allows students to describe, analyze, and interpret information through observing and discussing mind map images and movie scenarios [40].

Though the learning outcome effects were significant within both the intervention and control groups, the difference in learning outcomes between the 2 groups was also significant, suggesting the superiority of the QEMCL over mind mapping. This finding can be explained as follows: a picture may be worth a thousand words, but it cannot tell the whole story. Stories make the nurse-patient relationship and the context of using therapeutic techniques become apparent. Movie plots depict how clinical situations change and unfold over time. The mind mapping exercise may be able to stimulate visual thinking, but the QEMCL goes further by using storytelling to reinforce critical thinking. This narrative pedagogy helps students to think through and interpret situations they encounter from multiple perspectives and to understand clinical reasoning as a complex process [41,42]. With *Good Will Hunting*, the therapeutic relationship between Will Hunting and Dr Sean Maguire became vividly presented to students [ID#0107, 0115, and 0151]. The students learn how the therapist gets through the initial phase of the relationship with challenging situations from the patient’s mistrust [ID#0134]. Movie clips illustrate the effective use of techniques that open up opportunities for students to imitate and become accustomed to therapeutic communication [ID#0154 and 0156]. In addition, the clips provided thought-provoking ideas for students to reconsider prior misunderstandings and work on gaining new knowledge [ID#0134]. Finally, the clips directly expose students to diverse communication styles, teaching them to respect diversity and adapt their ways to suit culturally specific gestures [ID#0107 and 0142].

Previous studies support that dynamic visual media, such as videoclips, improve the processing and understanding of information by engaging both hemispheres of the brain. As the left side processes the dialogue, plot, rhythm, and lyrics, and the right side processes the visual images, sound effects, and harmonic relationships, videoclips capture students’ attention and stimulate thinking more quickly and more effectively than direct statements, setting out concepts, or straight course material [43]. Furthermore, compared to the noninteractive mind mapping exercise, interactive features of QEMCL such as stopping and replaying videoclips, getting feedback, and tracking personal progress allow students to personally distribute their attention and cognitive resources across the videos. The selected scenarios with embedded questions encourage students to think critically, correct their misunderstandings, and develop their own communication strategies. This participatory instructional strategy creates a constructive learning environment in which students are active participants who engage in problem-based and transformative learning. Students can advance at their own pace and in harmony with their unique cognitive skills and needs [44]. The QEMCL is, then, promising to facilitate procedural learning of discrimination, concept application, and rule-using, which are subskills of Bloom’s psychomotor domain [45].

Considering themes emerging from students’ reflection, those in the mind mapping group used keywords such as identifying key concepts, sorting those concepts into groups, placing them in classified orders, and linking them together to get a comprehensive picture. Those in the QEMCL group, on the other hand, used terms such as identifying proper concepts in a given scenario, differentiating among similar concepts, distinguishing principles applied in particular situations, clarifying the rationale for the choice of action, and discriminating between possible responses in various situations. Bloom’s taxonomy categorizes 6 levels of cognitive complexity: knowledge, comprehension, application, analysis, synthesis, and evaluation [45]. Students’ reflections indicated that the mind mapping exercise helped them memorize class content as organized structures and details, suggesting cognitive learning outcomes mostly at the knowledge level. Students in the other group stated that the QEMCL provides vivid examples of how to interpret patient behaviors and select different responses for the best probable outcome based on details unique to specific situations. Such reflection indicates that their learning outcomes were at the comprehension and application levels. This explanation is consistent with their higher posttest scores on the subscale of understanding (mean 8.53, SD 0.9; mean 7.41, SD 0.88) and applied knowledge (mean 8.77, SD 0.97; mean 7.47, SD 0.97), while the control group scores higher on the memorization subscale (mean 8.17, SD 1.05; mean 7.97, SD 0.9).

### Limitations

First, the knowledge test questions were identical for pre- and posttest, which could allow for familiarity with the questions. To minimize the testing effect, alternative measures with equivalent difficulty of the outcome variable should be used for repeated assessment. Second, even though the pretest score was statistically controlled as a covariate variable, other potential confounding variables such as prior academic performance in relevant courses should also be considered to ensure the validity of the results. Third, students in the second section who have the class from 10 AM to 12 PM may experience fatigue, reduced concentration, or cognitive overload, as they already had another class compared to those in the first section who had the class from 8 to 10 AM. Finally, the findings of this study have limited generalizability because the sample included nursing students from only one university. Therefore, further studies using nationwide systematic sampling are highly recommended.

### Conclusions

As instructional approaches are designed for varying purposes, identifying the best teaching strategies to promote nursing students’ engagement in academic and clinical settings has always been a challenge for nursing educators. While conventional text-based or lecture-based classroom environments facilitate students’ learning by listening, the mind mapping and the QEMCL exercises provide visual thinking tools that help students organize class content. The mind mapping exercise can help students organize their thoughts in a way that is easy to refer back to and build upon, while the QEMCL puts students in contact with realistic hypothetical situations and allows them to learn through observation, accumulating practical experience they will be able to integrate into real situations. Nursing education should not only promote students’ theoretical thinking but also improve their implicit knowledge and clinical skills. Incorporating computer-based drills and practice lessons with traditional class structures appears to be an effective pedagogical methodology, especially for lessons with limited class time. With the readiness of the university’s online support system and the simplicity of the application, the current learning program was implemented as planned with all students following study instructions. Given the easy identification of and access to appropriate material in this age of online learning, the use of the QEMCL program should be encouraged. Based on the current findings, we can conclude that QEMCL is both an enjoyable and effective teaching tool, yielding significantly higher learning gains than mind mapping exercises.

## Supplementary material

10.2196/71111 Checklist 1 CONSORT-eHEALTH checklist (V 1.6.1).

## Abbreviations

ANCOVA: analysis of covariance
CLEs: constructivist learning environments
IOC: item-objective congruence
QEMCL: Question-Embedded Movie Clips Learning
VTS: Visual Thinking Strategy
